# Circulating Biomarkers of Immune Activation Distinguish Viral Suppression from Nonsuppression in HAART-Treated Patients with Advanced HIV-1 Subtype C Infection

**DOI:** 10.1155/2014/198413

**Published:** 2014-04-06

**Authors:** Glen Malherbe, Helen C. Steel, Sharon Cassol, Tulio de Oliveira, Christopher J. Seebregts, Ronald Anderson, Edana Cassol, Theresa M. Rossouw

**Affiliations:** ^1^Medical Research Council Unit for Inflammation and Immunity, Department of Immunology, Faculty of Health Sciences, University of Pretoria, Pretoria 0001, South Africa; ^2^Africa Centre for Health and Population Studies, Mtubatuba 3935, South Africa; ^3^Jembi Health Systems NPC, Cape Town, Cape Town 7945, South Africa; ^4^School of Mathematics, Statistics and Computer Science, University of KwaZulu-Natal, Westville 3600, South Africa; ^5^Department of Cancer Immunology and AIDS, Dana Farber Cancer Institute, Boston MA02215, MA, USA

## Abstract

Few studies have examined immune activation profiles in patients with advanced HIV-1 subtype C infection or assessed their potential to predict responsiveness to HAART. BioPlex, ELISA, and nephelometric procedures were used to measure plasma levels of inflammatory biomarkers in HIV-1 subtype C-infected patients sampled before and after 6 months of successful HAART (n = 20); in patients failing HAART (n = 30); and in uninfected controls (n = 8). Prior to HAART, CXCL9, CXCL10, **β**2M, sTNF-R1, TGF-**β**1, IFN-**γ**, IL-6, TNF, and sCD14 were significantly elevated in HIV-1-infected patients compared to controls (*P* < 0.01). All of these markers, with the exception of sTNF-R1, were also elevated in patients failing HAART (*P* < 0.05). The persistently elevated levels of CXCL9, CXCL10, and **β**2M in patients failing therapy in the setting of a marked reduction in these markers in patients on successful HAART suggest that they may be useful not only to monitor immune activation during HAART, but also to distinguish between good and poor responders. In the case of sCD14 and TGF-**β**1, the levels of these biomarkers remained persistently elevated despite HAART-induced virological suppression, a finding that is consistent with ongoing monocyte-macrophage activation, underscoring a potential role for adjuvant anti-inflammatory therapy.

## 1. Introduction

In HIV-1 infection, depletion of T cells is caused by productive virus infection and Fas-mediated apoptosis of infected and uninfected cells [1, 2]. In addition, chronic immune activation, especially of cells of the innate immune system, together with accompanying, counteracting endogenous anti-inflammatory mechanisms, further contributes to T-cell depletion [3, 4]. These mechanisms include chronic activation of plasmacytoid dendritic cells and monocytes/macrophages. HIV infection of plasmacytoid dendritic cells causes persistent activation, resulting in excessive production of proapoptotic interferon (IFN)-*α*, as well as immunosuppressive indoleamine-2,3-dioxygenase and transforming growth factor (TGF)-*β* [4–13]. In the case of monocytes/macrophages, translocation of microbial products, especially lipopolysaccharide and DNA, across the damaged intestinal epithelium, results in persistent systemic activation of these cells due to interaction with Toll-like receptors 4 and 9, as well as with cytosolic pathogen nucleic acid sensors [14–23]. The resultant production of proinflammatory cytokines, especially TNF-*α*, drives T-cell activation and activation-induced cell death [6, 21, 22]. Sustained immune activation is associated with disease progression, AIDS, and death [24]. While highly active antiretroviral treatment (HAART) is able to suppress viral replication to levels of <25 copies/mL plasma and partially restore circulating CD4^+^ T cells, it is unable to normalize immune activation [21, 25].

Immune activation in HIV infection is associated with the presence of circulating proinflammatory/anti-inflammatory and antiviral cytokines/chemokines, as well as with other biomarkers of immune activation, which vary qualitatively and quantitatively with disease progression [26–31]. However, relatively little is known about the profile of circulating biomarkers of immune activation in the setting of advanced HIV-1 subtype C infection, as well as the usefulness of its measurement, not only in monitoring response to HAART, but also as a strategy to detect virologic treatment failure. These issues are the focus of the current study.

## 2. Methods

Black, adult (≥18 years) participants attending the Antiretroviral Clinic at a district hospital in Pretoria, South Africa, were included in this study. Ethics approval was granted by The Research Ethics Committee, Faculty of Health Sciences, University of Pretoria (Ethics Committee Approval number 46/2011). All participants gave informed consent and whole blood samples were collected in EDTA vacutainers, processed within 24 hours to separate the plasma component by centrifugation, and stored at −70°C for up to 37 months. CD4^+^ T-lymphocyte counts (CD4^+^) (Beckman Coulter SA (Pty) Ltd.) and HIV-1 RNA (VL) (Nuclisens HIV-1 Viral Load Assay v1.2 or v2.0) were measured by standard flow cytometric and PCR-based procedures respectively, according to manufacturer's instructions.

Sixty HIV-infected participants were followed from pre-treatment to approximately 6 months on HAART as part of a larger study on immune reconstitution inflammatory syndrome (IRIS). Pre-treatment samples were taken prior to the initiation of HAART in patients presenting with CD4^+^ counts ≤200 cells/*μ*L blood or WHO stage 4 disease. Twenty patients were randomly selected from those who started HAART, were clinically stable, did not develop clinical signs of IRIS during the first six months of treatment, and were virologically suppressed (VL < 50 copies/mL plasma) at approximately 6 months of HAART (suppressed group). Drug regimens consisted of two nucleos(t)ide reverse transcriptase inhibitors (NRTIs) (stavudine (d4T) + lamivudine (3TC), *n* = 18, or tenofovir (TDF) + 3TC, *n* = 2) and one nonnucleoside reverse transcriptase inhibitor (NNRTI) (efavirenz (EFV), *n* = 14 or nevirapine (NVP),  *n* = 4). Two patients were started on ritonavir-boosted lopinavir (LPV/r) for clinical reasons.

A second group consisted of 30 participants failing HAART as evidenced by two successive VL results of >1000 copies/mL plasma at least eight weeks apart despite intensive adherence counselling (failing group). Drug regimens consisted of two NRTIs (d4T + 3TC,  *n* = 23 or zidovudine (AZT) + 3TC, *n* = 7) and one NNRTI (EFV, *n* = 20 or NVP, *n* = 10). Participants had been referred for drug resistance testing and study samples were taken at the time of referral. They had been on HAART for a median time of 30 months (range 9–97 months) and had been failing treatment for a median of 15.5 months (range 5–38 months). Five patients (17%) had been referred from peripheral clinics and the duration of treatment failure could not be determined. Three patients (10%) had experienced treatment interruptions at some time before treatment failure and 13 (43%) never had a suppressed VL while on HAART. All patients with CD4^+^ ≤200 cells/*μ*L (*n* = 21) were on cotrimoxazole or dapsone prophylaxis.

A third group (*n* = 8) of black, HIV-uninfected, healthy control subjects was also included in the study. The median ages of the control, suppressed, and failing groups were 29 (range 24–49), 41.5 (25–63), and 40.5 (27–55) years, respectively, and the corresponding male : female ratios were 1 : 0.6, 1 : 4, and 1 : 4.

### 2.1. Circulating Biomarkers of Immune Activation

These were selected on the basis of being largely representative of T-cell, monocyte/macrophage, dendritic cell, and natural killer cell activation.

Circulating cytokines/chemokines were measured using (i) the Bioplex suspension bead array system (Bio-Rad Laboratories Inc., Hercules, CA, USA) (IL-6, IL-10, IFN-*γ*, TNF-*α*, CCL2/MCP-1, CCL3/MIP-1*α*, CCL4/MIP-1*β*, and CXCL10/IP-10) or (ii) conventional ELISA, namely, IFN-*α* (eBioscience Inc., San Diego, CA, USA); TGF-*β*1 total (Biolegend, San Diego, CA, USA); CXCL9/MIG and sTNF-R1 (Raybiotech Inc., Norcross, GA, USA); and sCD14 (Abcam, Cambridge, MA, USA). C-reactive protein (CRP) and *β*2-microglobulin (*β*2M) were assayed by nephelometry (Siemens healthcare Diagnostics, BN Prospec Nephelometer, Newark, USA). Previously published ranges for each of these parameters together with supporting references are shown as supplementary data (see Supplementary Material available online at http://dx.doi.org/10.1155/2014/198413).

### 2.2. Data Analyses and Statistics

As participant groups consisted of ≤30 individuals, data were considered to be nonparametric and distribution-free statistical tests implemented in Stata v11.2 (StataCorp). Continuous data were analysed according to the median, minimum, and maximum concentrations. Median concentrations of each parameter were compared between cohorts using the Wilcoxon Mann-Whitney test for independent groups and Wilcoxon signed rank sum test for matched groups. Correlations between parameters were determined using the Spearman correlation test for the HIV-infected pre-HAART group (*n* = 20), as well as for this group combined with the group failing HAART (*n* = 50). Statistical significance was set at *P* ≤ 0.05.

## 3. Results

### 3.1. Circulating CD4^+^ T-Lymphocyte Counts, HIV-1 Viral Loads, Cytokines/Chemokines, *β*2-Microglobulin, sCD14 and CRP in the Control, Suppressed (Pre- and Post-Therapy) and Failing Groups

CD4^+^counts, HIV-1 VL, and levels of inflammatory biomarkers are shown in Table 1. As expected, plasma VL decreased from a median of 53,000 to <50 RNA copies/mL plasma and there was a significant increase in the circulating CD4^+^ count (83 to 208 cells/*μ*L;  *P* < 0.0001) in the suppressed group. With respect to the circulating biomarkers of immune activation, CXCL9, CXCL10, TGF-*β*1, sTNF-R1, *β*2 M, and sCD14 were significantly elevated (*P* < 0.03) and CCL4 significantly decreased (*P* = 0.04) in the pre-HAART group relative to the control group, while IFN-*γ* was moderately increased but not significantly so (*P* = 0.07). Following 6 months of HAART, CXCL9, CXCL10, *β*2 M, IFN-*γ*, IL-6, TNF-*α*, and sTNF-R1 were significantly decreased (*P* < 0.01), CCL4 increased (*P* < 0.001), while TGF-*β*1 and sCD14 also remained elevated despite undetectable plasma VL. It is difficult to attribute major significance to the decreases in TNF-*α* and IL-6 as the pretherapy values for both were low. No difference was observed in IFN-*α*, CCL3, and CRP either between the HIV-infected and uninfected control group or the virologically suppressed group pre- and post-HAART.

In the failing group, the same 5 biomarkers (CXCL9, CXCL10, TGF-*β*1, *β*2M, and sCD14) were also significantly elevated compared with the control group (*P* < 0.02), the values for CXCL10 and *β*2M being somewhat lower than those of the pre-HAART group (*P* < 0.03), while those of CXCL9, TGF-*β*1, and sCD14 were essentially comparable (*P* > 0.5). Although the value for CCL2 was significantly lower and that of IL-10 higher than the corresponding values of the control group, interpretation is difficult as these values were low in both groups.

### 3.2. Analysis of Correlations between Variables

Correlations between CD4^+^, VL, and the various biomarkers in the pre-HAART group are shown in Table 2. CD4^+^ counts correlated negatively and significantly with VL and with sTNF-R1 and CCL2. Positive correlations were observed between VL and sCD14 and *β*2M. Significant positive correlations were also observed between several of the biomarkers including, but not limited to, IFN-*γ*, CXCL10, CCL2, CCL3, and TNF-*α*. Although not shown, correlations for the composite group (consisting of the pre-HAART and failing groups) were generally comparable, albeit weaker, with the exception of CD4^+^ count with VL (*r* = −0.64, *P* < 0.001), while the following modest correlations were found: (i) CCL4 with CXCL9, IL6, CCL3, and IFN-*γ* (*r* = 0.30, 0.43, resp.; *P* < 0.03,  *P* < 0.001); and (ii) *β*2M with CD4 counts, IL-6, and IFN-*γ* (*r* = −0.28,  0.43, resp.; *P* < 0.05,  *P* < 0.02).

## 4. Discussion

Our findings in patients infected with HIV-1 subtype C are consistent with the coexistence of distinct mechanisms of immune activation, which appear to be differentially affected by successful HAART [21, 25]. Although only moderately elevated pre-HAART, it is likely that IFN-*γ*, probably originating from CD4 and CD8 T cells, underpins the increases in CXCL9 and 10, a contention supported by the strong, positive intercorrelation between IFN-*γ* and CCL10, as well as that of CCL9 with CCL10. Other cell types such as dendritic cells and monocytes may also contribute to the increases in these cytokines pre-HAART following exposure of the cells to alternative activators such as IFN-*λ*1 [11, 32, 33]. The unexpectedly low level of IFN-*α*, as well as those of CCL2 and 3, may be due to advanced immunosuppression in the setting of high levels of TGF-*β*1 in this group of patients [34]. In the case of *β*2 M, CXCL 9 and 10, and TNF-R1 (a surrogate for TNF), HAART-associated decreases most likely reflect efficient viral suppression and consequent decreased turnover and reactivity of both CD4^+^ and CD8^+^ T cells.

The absence of effects of HAART on plasma sCD14, as previously reported by us and others [17, 21], as well as the increase in CCL4, is consistent with ongoing chronic inflammation due to sustained activation of monocytes/macrophages, even in the face of virally suppressive therapy, and may persist for several years [21, 35]. In this setting, the persistent activation of monocytes/macrophages, predominantly the subtype which coexpresess CD14 and CD16, is most likely driven by the process of microbial translocation [21, 24, 36]. The consequence is sustained generation of proinflammatory mediators and cytokine-driven T-cell death pathways. Interestingly, Sandler et al. recently reported significant positive correlations between plasma sCD14, IL-6, CRP, serum amyloid A, and D-dimer in patients infected with HIV-1 subtype B [37]. Subjects with the highest quartile of plasma sCD14 concentrations had a 6-fold higher risk of death than those in the lowest quartile [37].

In addition, supported by the findings of the current study, endogenous, monocyte/macrophage-targeted, anti-inflammatory mechanisms are also likely to contribute to ongoing immunosuppression with TGF-*β*1 appearing to play a pivotal role. Notwithstanding platelets, plasmacytoid dendritic cells, macrophages of the M2 phenotype, and immunoregulatory CD8^+^ T cells, immunosuppressive and profibrotic TGF-*β*1 is likely to originate predominantly from regulatory T cells [38, 39]. In this context it is noteworthy that extensive fibrosis of the T-cell zone of lymphoid tissue appears to be a significant factor in the failure of T-cell reconstitution following successful HAART [13].

Persistently elevated plasma levels of TGF-*β*1 and sCD14, even in the setting of ostensibly successful HAART, may therefore identify a subset of patients at highest risk of a poor outcome.

In the group of patients failing HAART, the circulating concentrations of CXCL9, CXCL10, and *β*2M were also significantly higher than those of the control group and, with the exception of CXCL9, significantly lower than the pre-HAART values for the suppressed group. The circulating concentrations of sCD14 and TGF-*β*1 in the failing group were comparable to those of the suppressed group both before and after therapy. Persistent elevations, or a rebound following an earlier decrease, in plasma CXCL9, CXCL10, and *β*2M appear to be associated with a poor response to HAART, suggesting that serial measurement of these biomarkers may be a useful adjunctive strategy. Nevertheless, measurement of VL clearly remains the definitive strategy in the clinical setting.

With respect to previous studies, our findings are generally in agreement with a recent study by Kamat et al. in which elevated circulating concentrations of CXCL9, CXCL10, sCD14, and soluble IL-2 receptor (sIL-2R) represented a profile which distinguished viremic and aviremic subjects infected with HIV-1 subtype B from uninfected, healthy control subjects [30]. In agreement with the report of Kamat et al. [30], we also detected a significant, negative correlation between numbers of circulating CD4^+^ T cells and VL but failed to show a correlation between these disease markers and CXCL10 in the pre-HAART group. However, this correlation was detected when the pre-HAART and failing groups were combined, most likely due to increased statistical power. As mentioned above, and in agreement with Kamat et al. [30] we also detected a significant positive correlation between CXCL9 and CXCL10, while in contrast to these authors, a significant, positive correlation between CXCL10 and IFN-*γ* was evident as can be expected in conditions of chronic inflammation.

Notwithstanding the different viral types investigated, several other important differences underscore the strengths of the current study. Most importantly, the profile of biomarkers of immune activation measured by Kamat et al. [30], which did not include *β*2M or TGF-*β*1, was not measured serially in a single cohort of patients pre- and post-HAART as done in the current study, which may account for the observed lack of effect of HAART on IFN-*γ* in the former study.

Limitations, however, are (i) small sample sizes; (ii) measurement of circulating biomarkers at a single time point (6 months) following initiation of HAART in the suppressed group; and (iii) no pretherapy measurement of circulating biomarkers prior to initiation of therapy in the failing group. Nonetheless, the general agreement with previous studies, predominantly in the setting of HIV-1 subtype B infection, supports the reliability of our findings.

In conclusion, successful administration of HAART to patients with HIV-1 subtype C infection is accompanied by significant decreases in circulating biomarkers associated with T-cell activation and turnover (IFN-*γ*, CXCL9, CXCL10, sTNF-R1, and *β*2M). Serial measurement of 3 of these (CXCL9, CXCL10, and *β*2M) may represent a useful adjunct to measurement of viral loads in monitoring responses to HAART. In addition, persistently elevated levels of sCD14 and TGF-*β*1, despite successful HAART, are consistent with chronic activation of monocytes/macrophages and possible risk of a poor outcome, underscoring the adjunctive therapeutic potential of monocyte/macrophage-targeted anti-inflammatory chemotherapy in patients with advanced HIV infection.

## Supplementary Material

This is a list of previously published studies documenting normal ranges for the various circulating biomarkers of immune activation assayed in the current study.

## Figures and Tables

**Table 1:** CD4^+^ T-cell lymphocyte counts, HIV-1 viral loads, and circulating biomarkers of immune activation, in the 3 study groups.

Measurement	HIV Negative, *n* = 8		Suppressed group, *n* = 20				Failing group, *n* = 30	
			Pre-HAART		Post-HAART			
	Median	Range*	Median	Range*	Median	Range*	Median	Range*
CD4^+^ T-cell count	N/A	N/A	83	3–296	208^#^	86–537	126^#^	10–533
Viral load	N/A	N/A	53 000	5700–330 000	<50^#^		7300^#^	240–350 000
sCD14	4116.7	3185.7–5169.7	8815^+^	4945.1–11 000	8259.1^+^	3424.7–11 000	9209.8^+^	430.5–11 000
*β*2-microglobulin	1.6	1.3–2.4	4.8^+^	2.0–8.6	2.5^+#^	1.7–3.6	3.3^+#^	2–9.1
CRP	4.0	0–15.1	1.8	0.1–43.8	3.9	0.3–30.2	3.2	0.5–97.3
TGF-*β*1 Total	6458.8	1845.8–12 448.1	12 701.9^+^	4634.8–37 587.2	14 882.2^+^	4426.7–50 000	11 399.3^+^	0–43 434.5
IFN-*γ*	18.2	0–210.4	53.1	4.6–392.8	16.9^#^	0–74.4	40.9	0–403.1
CXCL10	416.1	225.7–688.5	1864.0^+^	858.3–66 740.7	822.5^+#^	212.2–1378.0	1097.0^+#^	150.2–3957.1
CXCL9	849.1	246–2991.8	6059.5^+^	1714.9–12 000	2940.6^+#^	1410.2–12 000	4849.4^+^	1 318–12 000
IFN-*α*	18.0	16–28.2	19.4	11.7–64.9	16.8	14.3–54.7	17.3	14.3–120.1
IL-6	1.5	0.5–11.3	2.7	0.9–40.6	1.2^#^	0.3–4.0	2.3	1.1–32.3
IL-10	0.9	0.4–14.9	2.5	0.5–26.2	1.4	0.6–2.6	2.2^+^	0.8–9.6
CCL2	20.3	9.6–28.0	13.5	2.3–33.2	8.3^+^	3.8–32.2	6.5^+#^	1.4–46.9
CCL3	2.6	2.3–5.8	2.9	2.1–12.8	2.8	2.3–11.5	2.8	2.3–32.1
CCL4	39.8	23.5–70.4	22.8^+^	0–82.0	48.8^#^	7.6–84.1	33.9	4.4–110.3
TNF-*α*	0	0–36.5	1.1	0–124.0	0^#^	0–8.5	1.7	0–223.6
sTNF-R1	353.2	190.9–470.6	512.2^+^	212.3–800	380.7^#^	214.5–635.7	363.5	151.3–800

All results are presented as pg/mL with the exception of sCD14 (ng/mL), *β*2-microblobulin and CRP (µg/mL), circulating CD4^+^ T cell counts (cells/µL blood), and viral loads (copies/mL).

*Range: minimum to maximum value.

^+^ *P* < 0.04–*P* < 0.001 for comparison with corresponding values for healthy control subjects.

^#^ *P* < 0.02–*P* < 0.001 for comparison with corresponding pre-HAART values.

Median concentrations were compared by means of Wilcoxon Mann-Whitney test for independent groups and Wilcoxon signed rank sum test for matched groups.

**Table 2:** Most significant correlations in the HIV-infected pre-HAART group [*n* = 20] (paired values represent the correlation coefficients with the corresponding *P* values in parentheses).

	VL	TNF-R1	sCD14	CXCL9	IL-6	IL-10	IFN-*γ*	CXCL10	CCL2	CCL3	CCL4	TNF-*α*	CRP	*β*2M
CD4	**−0.45 (0.05)**	**−0.52 (0.02)**	−0.25 (0.28)	−0.04 (0.88)	−0.32 (0.17)	−0.27 (0.25)	−0.23 (0.33)	−0.18 (0.45)	**−0.59 (0.01)**	−0.28 (0.23)	−0.30 (0.20)	−0.13 (0.57)	−0.42 (0.07)	−0.30 (0.20)
VL	1.00	0.27 (0.25)	**0.58 (0.01)**	0.25 (0.28)	0.37 (0.10)	0.07 (0.78)	0.20 (0.40)	0.40 (0.08)	0.36 (0.12)	0.29 (0.22)	0.29 (0.21)	−0.06 (0.79)	0.22 (0.35)	**0.64 (0.00)**
IFN-*α*	0.31 (0.18)	0.33 (0.16)	**0.58 (0.01)**	0.31 (0.18)	**0.58 (0.01)**	0.26 (0.27)	**0.44 (0.05)**	0.14 (0.56)	0.20 (0.40)	**0.66 (0.00)**	−0.07 (0.77)	**0.45 (0.05)**	0.19 (0.42)	0.30 (0.20)
TNF-R1	0.27 (0.25)	1.00	**0.58 (0.01)**	0.06 (0.79)	**0.46 (0.04)**	0.29 (0.21)	0.06 (0.79)	0.04 (0.88)	0.03 (0.90)	0.25 (0.29)	−0.03 (0.90)	−0.05 (0.88)	**0.54 (0.01)**	0.39 (0.09)
sCD14	**0.58 (0.01)**	0.58 (0.01)	1.00	**0.45 (0.05)**	**0.80 (0.00)**	0.19 (0.43)	0.43 (0.06)	0.37 (0.10)	0.11 (0.60)	**0.63 (0.00)**	0.19 (0.44)	0.26 (0.28)	**0.44 (0.05)**	**0.62 (0.00)**
CXCL9	0.25 (0.28)	0.06 (0.79)	**0.45 (0.05)**	1.00	**0.50 (0.02)**	0.21 (0.37)	**0.64 (0.00)**	**0.68 (0.00)**	0.19 (0.41)	**0.64 (0.00)**	0.17 (0.46)	**0.48 (0.03)**	0.25 (0.30)	**0.67 (0.00)**
IL-6	0.37 (0.10)	**0.46 (0.04)**	**0.80 (0.00)**	**0.50 (0.02)**	1.00	**0.50 (0.03)**	**0.74 (0.00)**	0.41 (0.08)	0.37 (0.11)	**0.83 (0.00)**	0.36 (0.11)	**0.57 (0.01)**	0.38 (0.10)	0.42 (0.06)
IL-10	0.07 (0.78)	0.19 (0.21)	0.19 (0.43)	0.21 (0.37)	**0.50 (0.03)**	1.00	**0.50 (0.03)**	0.20 (0.41)	0.34 (0.14)	**0.66 (0.00)**	0.43 (0.06)	0.39 (0.09)	−0.12 (0.62)	0.16 (0.49)
IFN-*γ*	0.20 (0.40)	0.06 (0.79)	0.43 (0.06)	**0.64 (0.00)**	**0.74 (0.00)**	**0.50 (0.03)**	1.00	**0.68 (0.00)**	**0.60 (0.01)**	**0.79 (0.00)**	0.42 (0.06)	**0.64 (0.00)**	0.06 (0.80)	0.35 (0.13)
CXCL10	0.40 (0.08)	0.04 (0.88)	0.37 (0.10)	**0.68 (0.00)**	0.41 (0.08)	0.20 (0.41)	**0.68 (0.00)**	1.00	0.36 (0.12)	**0.48 (0.03)**	0.37 (0.11)	0.37 (0.11)	0.25 (0.30)	**0.60 (0.05)**
CCL2	0.36 (0.12)	0.03 (0.90)	0.11 (0.63)	0.19 (0.41)	0.37 (0.11)	0.34 (0.14)	**0.60 (0.01)**	0.36 (0.12)	1.00	**0.48 (0.03)**	**0.58 (0.01)**	0.28 (0.23)	0.04 (0.89)	0.22 (0.35)
CCL3	0.29 (0.22)	0.25 (0.29)	**0.63 (0.00)**	**0.64 (0.00)**	**0.83 (0.00)**	**0.66 (0.00)**	**0.79 (0.00)**	**0.48 (0.03)**	**0.48 (0.03)**	1.00	0.41 (0.07)	**0.64 (0.00)**	0.12 (0.61)	**0.44 (0.05)**

Spearman correlation test; statistically significant correlations (*P* ≤ 0.05) are shown in bold.
