# Supplementary material for: Circulating Biomarkers of Immune Activation Distinguish Viral Suppression from Nonsuppression in HAART-Treated Patients with Advanced HIV-1 Subtype C Infection
Source: Mediators Inflamm. 2014 Apr 6;2014:198413. doi: 10.1155/2014/198413 (PMC3997875; doi:10.1155/2014/198413)
Supplement: Supplementary file 1 — This is a list of previously published studies documenting normal ranges for the various circulating biomarkers of immune activation assayed in the current study. [file 198413.f1.pdf]

Supplementary Table 1: Reference ranges of inflammatory cytokines in healthy individuals as described in the literature

	Units	Reference Range
**sCD14**	ng/ml	2200 – 18300[1]; 1500 – 1900[2]
**β2-microglobulin**	mg/l	1.09 – 2.53[1]
**CRP**	mg/l	< 10[1]
**TGF-β1 total**	pg/ml	1690.0 – 41250.0[1]; 30.9 – 65.1[3]
**IFN-γ**	pg/ml	0.00[4]
**CXCL10**	pg/ml	202 – 1480[5] ; 232 – 1019[6]
**CXCL9**	pg/ml	37 – 463[6]
**IFN-α**	pg/ml	0 – 75.7[1]
**IL-6**	pg/ml	0 – 3.3[7]; 1.56 – 8.6[8]; 0 – 3.1[9]
**IL-10**	pg/ml	3.9 – 8.8[8]; 0 – 8.0[9]
**CCL2**	pg/ml	29.2 – 156.4[10]; 18 – 152[6]
**CCL3**	pg/ml	0 – 208[11]
**CCL4**	pg/ml	1 – 41[11]
**TNF-α**	pg/ml	0 -6.3[9]
**sTNF-RI**	pg/ml	583 – 804[12]; 3700 – 3950[13]; 1037.7 – 1632.1[14]

1. Abcam human CD14 ELISA kit protocol. Available at: http://www.abcam.com/CD14-Human-ELISA-Kit-ab46541.html Accessed 08 May 2013.

2. Bas S, Gauthier BR, Spenato U, Stingelin S, Gabay C. 2004. CD14 is an acute phase protein. Journal of Immunology **2004**; 172(7):4470-9.

3. Lebrecht A, Grimm C, Euller G, et al. Transforming growth factor beta 1 serum levels in subjects with preinvasive and invasive lesions of the breast. International Journal of Biological Markers **2004**; 19(3):236-9.

4. Pisa P, Stenke L, Bernell P, Hansson M, Hast R. Tumor necrosis factor-alpha and interferon-gamma in serum of multiple myeloma subjects. Anticancer Research **1990**; 10(3):817-820.

5. Lee N, Wong CK, Chan PKS, et al. Acute encephalopathy associated with influenza A infection in adults. Emerging Infectious Diseases **2010**; 16(1):139-142.

6. Ng PC, Lam CWK, Li AM, et al. Chemokine response in children with SARS. Archives of Disease in Childhood **2005**; 90:422-3.

7. Knudsen LS, Hetland ML, Johansen JS, et al. Changes in plasma IL-6, plasma VEGF and serum YKL-40 during treatment with etanercept and methotrexate or etanercept alone in subjects with active rheumatoid arthritis despite methotrexate therapy. Biomarker insights **2009**; 4:91-5.

8. Kozłowski L, Zakrzewska I, Tokajuk P, Wojtukiewicz MZ. Concentration of interleukin-6 (IL-6), interleukin-8 (IL-8) and interleukin-10 (IL-10) in blood serum of breast cancer subjects. Annales Academiae Medicae Bialostocensis **2003**; 48:82-4.

9. Theusinger OM, Baulig W, Seifert B, Emmert MY, Spahn DR, Asmis LM. Relative concentrations of haemostatic factors and cytokines in solvent/detergent-treated and fresh-frozen plasma. British Journal of Anaesthia **2011**; 106(4):505-511.

10. Berrahmoune H, Lamont VJ, Herbeth B, FitzGerald PS, Visvikis-Siest S. Biological determinants of and reference values for plasma interleukin-8, monocyte chemoattractant protein-1, epidermal growth factor, and vascular endothelial growth factor: Results from the STANISLAS cohort. Clinical Chemistry **2006**; 52: 504-510.

11. van Breemen MJ, de Fost M, Voerman JS, et al. Increased plasma macrophage inflammatory protein (MIP)-1alpha and MIP-1beta levels in type 1 Gaucher disease. Biochimica & Biophysica Acta **2007**; 1772(7):788-796.

12. Parsons PE, Matthay MA, Ware LB, Eisner MD, and the National Heart, Lung, Blood Institute Acute Respiratory Distress Syndrome Clinical Trials Network. Elevated plasma levels of soluble TNF receptors are associated with morbidity and mortality in subjects with acute lung injury. American Journal of Physiology - Lung Cellular & Molecular Physiology **2005**; 228:L426-L431.

13. Tziakas D, Chalikias G, Parissis JT, et al. Prolonged activation of tumor necrosis factor (TNF)-alpha and its soluble receptors in chronic heart failure subjects both in the compensated and decompensated state. Interplay between their levels and metalloproteinase-3. European Cytokine Network **2004**; 15(3):231-9.

14. Lin SY, Wang YY, Sheu WHH. Increased serum leptin concentrations correlate with soluble tumor necrosis factor receptor levels in subjects with cirrhosis. Clinical Endocrinology **2002**; 57:805-811.
